# QUALITY OF CARE INTERACTIONS IN ACUTE CARE

**DOI:** 10.1093/geroni/igad104.1109

**Published:** 2023-12-21

**Authors:** Rachel McPherson

**Affiliations:** University of Maryland, Baltimore, Maryland, United States

## Abstract

Care interactions are verbal or nonverbal exchanges between staff and patients and promote quality of care and optimize outcomes for hospitalized patients living with dementia. Care interactions vary regarding quality and are differentiated between positive interactions (e.g., interactions that provide beneficial companionship or appropriate care communication for residents), neutral interactions (e.g., brief, indifferent interactions), or negative interactions (e.g., interactions that restrict residents due to safety concerns or interactions that are restrictive for no justified reason). Patients exposed to negative care interactions, particularly those that are living with dementia, are at risk for negative outcomes such as increased resistiveness to care, anxiety, depression, and apathy. The Quality of Care Interactions Survey (QuIS) is an observation measure used to evaluate interactions between staff and patients based on definitions of positive social, positive care, neutral, negative protective, or negative restrictive interaction. Scores range from 0 to 7, with higher scores indicating more positive care interactions between staff and residents. Nursing interactions with the first 365 patients in the FFC-AC-EIT study indicated that on admission the majority of the care interactions were positive (82%), half were neutral (50%), and a small percentage were negative protective (7%) and negative restrictive (6%). By discharge, there was a decrease in neutral interactions to 47%, negative protective to 6% and negative restrictive to 5%. Ongoing oversight and education is needed to decrease neutral and negative interactions between staff and patients using techniques provided in our function focused care intervention.

